# Validation and test-retest reliability of the Royal Free Interview for Spiritual and Religious Beliefs when adapted to a Greek population

**DOI:** 10.1186/1744-859X-4-6

**Published:** 2005-03-04

**Authors:** Despina Sapountzi-Krepia, Vasilios Raftopoulos, Marcos Sgantzos, Evangelia Kotrotsiou, Zoe Roupa-Darivaki, Kalliope Sotiropoulou, Ioanna Ntourou, Alexandra Dimitriadou

**Affiliations:** 1 Nursing Department, Technological Educational Institute of Thessaloniki, Thessaloniki, Greece; 2 Hellenic Centre for Infectious Diseases Control, Athens, Greece; 3 Medical School, University of Thessaly, Larissa, Greece; 4 Nursing Department, Technological Educational Institute of Larissa, Larissa, Greece; 5 Health Services of The National Bank of Greece, Athens, Greece; 6 University of Cologne, Faculty of Remedial Sciences, Cologne, Germany

## Abstract

**Background:**

The self-report version of the Royal Free Interview for Religious and Spiritual Beliefs has been confirmed as a valid and reliable scale, assessing the manner and nature in which spiritual beliefs are expressed. The aim of the present study was to evaluate the test-retest reliability and psychometric properties of the Greek version of the Royal Free Interview for Religious and Spiritual Beliefs.

**Methods:**

A total of 209 persons (77 men and 132 women) with a mean age of 28.33 ± 9.44 years participated in the study (test group). We subsequently approached 139 participants of the test group with a mean age of 28.93 ± 9.60 years, who were asked to complete the Royal Free Questionnaire a second time two weeks later (retest group).

**Results:**

The vast majority of participants (58.9%) reported both a religious and a spiritual belief, compared to 52 (25.1%) who told of a religious belief only. The internal consistency of the spiritual scale for the test group proved to be good, as standardized inter-item reliability / Cronbach's alpha was 0.83. Item-total correlations ranged from 0.51 to 0.73. They indicated very good levels of differentiation, thus showing that the questions were appropriate. Internal consistency of the spiritual scale for the retest group proved as good as for the test group. Standardized inter-item reliability / Cronbach's alpha was 0.84. Item-total correlations ranged from 0.52 to 0.75. The Pearson correlation coefficient for the total test-retest score of the spiritual scale was 0.754 (p < 0.001).

**Conclusion:**

The Greek version of the Royal Free Interview for Religious and Spiritual Beliefs is reliable and thus suitable for use in Greece.

## Background

Religious faith, spirituality and spiritual beliefs were rarely discussed in the psychological or medical literature of the '80s [1,2]. However, over the past decade an increasing interest on the part of health care professionals in spirituality and spiritual care emerged, and related articles were frequently published in medical and nursing journals [3-8]. Religious faith and spirituality are now widely recognized as important components of subjective human wellness [9], of health care outcomes [10-14], of holistic nursing care [15,16] and of the quality of hospital care [17-19].

Nevertheless, the terms spirituality and religiousness have been used in different ways by different authors, sometimes interchangeably because of the elusiveness of both concepts. Narayanasamy argues that the lack of any authoritative definition of these two terms has resulted in a multiple definition and hence in the confusion surrounding these concepts [20]. Furthermore, Mokuau et al. aptly state: *"...the difficulties in developing standardized definitions and measures relate to varying interpretations of religiousness and spirituality..." *and they stress that the potential for providing quality care that integrates religiousness and spirituality to a large extent depends upon the development of measures that are at least psychometrically sound [21].

Studies on religiousness, spirituality and spiritual care are rare in Greece [17,22] and this may be the reason for the present lack of a valid instrument. Sapountzi et al. mention that *"...the lack of valid instruments is a widespread phenomenon, concerning not only Greece but rather all non-English speaking countries, owing to the fact that in this day and age, English is the language of science, just as Greek was in biblical times..." *[23].

In 1995 King et al. developed a scale, "The Royal Free Interview for Religious and Spiritual Beliefs", in order to evaluate religious and spiritual beliefs in a variety of populations. Some years later, this scale was modified in order to make it more functional [24,25]. The Royal Free Interview for Religious and Spiritual Beliefs is a valid and reliable scale; in the English version [24], it is short and simple and can be easily completed by most people. Moreover, it focuses on the strength and the consequences of faith, rather than on the specific nature of each belief and as King et al. stress, the *"...interview was designed specifically to avoid a focus on any one religious system or type of spiritual belief and thus comparisons were impossible to make..." *[24].

At the time that The Royal Free Interview for Religious and Spiritual Beliefs was published, the researchers of the present paper were looking for an instrument capable of measuring spirituality and religiousness in the Greek population [24]. Following careful consideration, they decided to translate an already valid instrument instead of developing a new one, choosing a European rather than an American one, because they believe that although cultural differences exist among European populations, they also share many common cultural issues. In addition, taking into account that the vast majority of the population living in Greece belongs to the Greek Orthodox Church, it was desirable to find a scale designed specifically to avoid a focus on any one religious system or type of spiritual belief. The Royal Free Interview for Religious and Spiritual Beliefs fulfilled most of the criteria set, and therefore the researchers approached Professor King, asking for his permission to translate the scale into the Greek language and validate it. After obtaining written permission, the Royal Free Interview for Religious and Spiritual Beliefs was translated into Greek in 2003 [23]. In a second stage, the research team continued evaluating the psychometric properties of the scale. Let us point out that as a result of extensive discussions among the research team and with other researchers, we decided to revise the question "How would you describe yourself? (tick one or more)". More precisely, the modified question in the Greek version asked participants to tick a specific part of Greece as their place of origin. Given that over the past twenty years, Greece has been faced with immigration from the Balkan countries and the former USSR, the question was adapted to include these newcomers' origin as well. The question was thus revised, and instead of asking respondents to indicate specific parts of Greece as their place of origin, they are now requested to indicate their origin from an international perspective. The modification was made in order to make the question suitable for people from a variety of ethnic backgrounds living in contemporary Greece.

This paper reports on test – retest reliability and the psychometric properties of the Greek version of the Royal Free Interview for Spiritual and Religious Beliefs.

## Methods

Cross-cultural validation of an existing scale, such as the Royal Free Interview for Spiritual and Religious Beliefs, has the great advantage of avoiding the initial stages of development of a new questionnaire, which is a lengthy process [26]. Furthermore, translation and adaptation of a scale into different languages makes it possible to use the questionnaires in comparative international multi-center studies. This is why we decided first to translate, re-translate and then proceed to check the validity, reliability and psychometric properties of the scale for a Greek population [27-29].

The translation and the cultural adaptation of the Royal Free Interview for Spiritual and Religious Beliefs was carried out at an early stage [23]. A panel of experts who were also bilinguals was requested to translate the English version of the Royal Free questionnaire. Furthermore, in order to assess the linguistic accuracy of the translated instrument, a pilot study using bilingual persons was carried out. Details related to the translation and adaptation of the Royal Free questionnaire for spiritual and religious beliefs are mentioned elsewhere [23].

### Test – Retest

To evaluate the stability of a translated instrument, it is recommended that it be tested in the target culture based on a test-retest design [30]. It is difficult to establish standards for retest reliability since many factors need to be considered, such as the time between pre-test and post-test, learning obtained from the pre-test or between tests, and the type of test (trait or state). Some researchers believe that it is sufficient to know that the retest coefficient is statistically significant from zero, although Huck & Cormier have warned against such use [31]. The Spearman Rank Correlation Coefficient (rho) between scores produced at the first and second testing was calculated to assess the test-retest reliability. However, the calculation of correlation coefficients is not a sufficient method to test reliability and reproducibility of a method and its results, because it is an index of correlation and not an index of agreement [32-34]. There is less agreement about intra-class correlation coefficients. For questions with categorical responses, such as questions 1 and 12 to 14, kappa statistics were used. Face validity and content validity were established during the stage of translation and modification of the scale into Greek [23].

### Data analysis

All items were coded and scored, and the completed questionnaires were included in the data analysis set. Individual unanswered items were excluded from the analysis. Statistical Package for Social Sciences 10.0 computer software was used for the statistical analysis of the data obtained [35]. The Pearson correlation coefficient was used to calculate the linear correlation of two continuous variables. The Chi-squared test was used between two nominal variables. The *t*-test assessed whether the means of two groups were statistically different from each other. Values less than 0.05 were considered statistically significant, unless otherwise stated.

### Sample

Potential subjects meeting the following inclusion criteria were selected to participate in the study: (1) willing to participate, (2) over 18 years of age (3) capable of speaking and reading Greek and (4) no cognitive impairment, according to the research team's assessment. Potential subjects were recruited from the community on the basis of their availability. They received a brief explanation of the purpose and the aim of the study, and those who agreed to participate were asked to sign an informed consent form. We finally approached 209 people (77 men and 132 women) informally through hospitals and institutions of learning. Participants were divided in two major groups: *Group A: *209 participants who answered to the Royal Free questionnaire for the first time, thus called the test group and *Group B: *139 participants who were given the Royal Free questionnaire a second time two weeks later (the retest group). Group A was the test group and the group B was the retest group. The mean age of group A was 28.33 ± 9.44 and that of group B was 28.93 ± 9.60 years.

In the test group, there was a statistically significant difference between the two genders and their mean age, with women being younger than men [26.73 (sd 9.02) cf 31.08 (sd 9.56), mean diff 4.34, CI 1.69 to 7.00, df (unequal variance) 151.707, p = .001]. A similar difference was observed in the retest group [26.97 (sd 9.25) cf 30.82 (sd 9.41), mean diff 3.86, CI 0.46 to 7.25, df (unequal variance) 86.778, p = .027]. Participants in both groups were mainly white, single students. Table 1 shows the distribution of the sample according to demographic characteristics.

**Table 1:** Demographic characteristics of the sample

	**Group A**		**Group B**	
	
	**N**	**%**	**N**	**%**
***Gender***				
Male	77	36.8	46	33.3
Female	132	63.2	92	66.7
				
***Marital status***				
Married	43	20.8	31	22.5
Cohabiting	35	16.9	22	15.9
Divorced	6	2.9	4	2.9
Separated	6	2.9	4	2.9
Single	117	56.5	77	55.8
				
***Ethnic group***				
White Europeans from EU	198	96.1	131	95.6
Russian	4	1.9	2	1.5
White Europeans from Eastern Europe countries out of EU	3	1.5	3	2.2
Albanian	1	0.5	1	0.7
				
***Occupation***				
Employed	51	28.2	33	28.5
Unemployed seeking work	17	9.4	12	10.3
Student	101	55.8	63	54.3
Retired	1	0.6	1	0.9
Home manager	11	6.0	7	6.0

## Results

The majority of participants in both groups were Orthodox Christians (for group A: N = 106, 62.4% for group B: N = 68, 58.6%) as compared to smaller percentages of those with no religious faith (for group A: N = 5, 2.9% for group B: N = 4, 3.4%) Roman Catholics (for group A: N = 19, 11.2% for group B: N = 12, 10.3%) Protestants (for group A: N = 18, 10.6% for group B: N = 14, 12.1%) Sunni Muslims (for group A: N = 1, 0.6% for group B: N = 1, 0.9%) and those belonging to other religions (for group A: N = 21, 12.4% for group B: N = 17, 14.7%).

A total of 18 out of 207 participants of the test group (8.7%) stated that they had no religious or spiritual understanding of their life; 44 (21.1%) reported a religious belief; 18 (8.7%) told of a spiritual belief and the vast majority of participants (61.4%) reported both a religious and a spiritual belief. In group B, responses were similar. Those with a spiritual or religious understanding of their life explained that they believed in God, the Saints, the Holy Trinity, a superior creature, or the Bible.

Spiritual belief differed in a statistically significant manner between the two genders of the test population (x^2 ^= 25.808, df = 3, p < .001) as shown in table 2. The vast majority of women (66.8%), rather than men (41.4%), reported religious and spirituals beliefs and only 2.1% of the women and 12.6% of the men had neither religious nor spiritual beliefs. Logistic regression analysis indicated that women (OR 2.3, 95%CI 1.1–4.6) were significantly more likely to hold a religious and spiritual view of life. Table 2 presents the gender-specific difference of mean scores of spiritual scale.

**Table 2:** Difference of mean scores of spiritual scale between two genders

**Question**	**Men**		**Women**		
		
	**N**	**M (SD)**	**N**	**M (SD)**	**t-test (Sig.)**
Q3: strength of belief	83	6.95 (2.21)	183	7.10 (2.12)	.593
Q7: practice of faith	83	4.94 (2.87)	180	5.91 (2.64)	.008
Q8: influence of power or force	85	5.36 (3.14)	182	6.66 (2.38)	<.001
Q9: enable you to cope	85	5.44 (3.10)	180	6.72 (2.29)	<.001
Q10: influence on world affairs	85	3.48 (2.90)	181	4.69 (2.77)	.001
Q11: natural disasters	85	4.26 (3.27)	182	5.45 (2.93)	.003

Four persons (1.5%) answered that they underwent an intense experience at a time when they almost died, but were eventually revived. Four other persons were uncertain as to whether or not they had had such an experience. For these four persons, the mean effect of this experience on their lives was moderate (4.69 ± 2.9). The majority of persons participating in our study answered that they prayed by themselves (N = 215, 77.3%) or with others (N = 24, 8.6%). Only 57 persons (20.5%) stated that religious ceremonies play a central role in their faith; 43.2% said the same of meditation, 60.8% of reading and study, 36.3% of contact to religious leaders and 7.2% none of the above.

After we tallied the scores of participants for questions 3, 7 to 11, which make up the spiritual scale, we found that during the test phase the t-test was significant (t = -3.562, df = 256, p < .001) and showed that the mean scores of the participants on the spiritual scale differed significantly between women (mean = 36.10 ± 10.54) and men (mean = 31.80 ± 11.25), as shown in table 2. The retest did not lead to the same result, as the t-test showed no statistical difference between the two genders (p = .563). There was a gender-specific difference (Fisher's exact test p = .001), though, between those who believed that prayer plays an important role in their faith: the majority of women (75.6%) like to pray alone, instead of men (60.9%) who prefer to pray with others.

One hundred and sixty-five participants (62%) answered that they do not communicate with a spiritual power at all, i.e. by way of prayer or contact via a medium. Eleven persons (4.1%) were uncertain. Women communicate more often by praying than do men (Chi-Square test 7,595 df = 2 p = .022). Twenty-five percent of the men, as against 45% of the women communicate with a spiritual power in some other way.

The percentage of those who believe that we continue to exist in some form after death (37.5%) was similar to those who were uncertain (39.3%). Thirty-two participants (23.9%) had had an intense experience through which they sensed some deep new meaning in life that lasted for a few moments, hours or even days. Eleven of them felt it once (44%) and ten of them had had eleven to fourteen such intense experiences (40%). This experience lasted for 7.96 ± 8.53 days. For some, it lasted 4.88 ± 0.61 hours, for others 5.02 ± 0.65 minutes and for a few individuals it lasted for 5 seconds.

When asked to describe this experience, they answered that the incident was a prophetic dream, the recovery from an accident or an illness, the presence of an invisible force, the birth of their child, a detachment of the material body, a strong feeling of happiness, of feeling protected from harm. Further analysis of this open question (describe the intense experience) put to those participants who had had an intense experience rendered a description of a dreamy journey to an unknown place, filling them with a strong euphoric feeling of happiness, wellness and inner peace. The main characteristic of this experience was the lack of any sense of control over the circumstances. They felt no fear, although it was an unusual experience – perhaps precisely because it was a perceived spiritual experience, providing an opportunity to become closer to God or clarifying the grace of God. It was an isolated moment of physical and bodily sensation, or a sharing emotion. For others it was a unique and private emotional experience, or simply a prophetic reassurance of future recovery from a difficult situation. Some of the respondents were not able to describe it, even though their experience had been very intense.

Between those 90 participants (67.2%) who had never felt an intense experience, two persons mentioned that they had undergone an intense experience at a time when they almost died but were eventually revived. It was an incident that changed their lives.

Fourteen (88.9%) of the 18 participants with a spiritual belief communicate in some way with a spiritual power, compared to 26 (59.1%) of the 44 persons with a religious faith and 67 (52.8%) of the 127 persons with both a religious and a spiritual belief (Chi Squared 18.20, df = 6, p = .006). Respondents who reported having intensely experienced a power were more likely to believe in a spiritual power that influences the universe (5.52 ± 3.30) than those who had never had such an experience (4.25 ± 2.54). Belief in the existence of some form of life after death was more common among Muslims and those who do not belong to a specific religion. The majority of Orthodox Christians did not believe in life after death (73.6%). Forty-nine (38.9%) of the 126 persons who spoke of both a religious and spiritual faith believed in life after death, as against forty-five (35.7%) persons who were uncertain.

There was a major difference in mean scores on the spiritual scale between persons who expressed a religious/spiritual view of life on the Royal Free Questionnaire and those who did not [37.2 (sd 10.2) cf 23.1 (sd 18.3), mean diff 14.0, CI 6.6 to 21.5, df (equal variance) 131, p < 0.0001]. Those who communicate with a spiritual power obtained higher mean scores on the spiritual scale than those who do not [42.4 (sd 8.9) cf 31.1 (sd 9.7), mean diff 11.3, CI 8.5 to 14.1, df (unequal variance) 156.55, p < 0.0001]. Women expressed a higher spirituality than did men [37 (sd 12) cf 31 (sd 10.7), mean diff -5.97, CI -9.4 to -2.5, df (unequal variance) 125,64, p < 0.0001].

### Internal consistency of the spiritual scale and test – retest reliability

The internal consistency of the spiritual scale for the test group proved to be good, as standardised inter-item reliability / Cronbach's alpha was 0.83. This is above the accepted limit of 0.70 [35]. Item-total correlations ranged from 0.51 to 0.73. They indicated very good levels of differentiation, thus showing that questions were appropriate. Internal consistency of the spiritual scale for the retest group proved as good as for the test group. Standardized inter-item reliability / Cronbach's alpha was 0.84. Item-total correlations ranged from 0.52 to 0.75.

Kappa statistics for categorical items are summarized in table 3. Kappa is a chance-corrected measure of agreement, as it represents the proportion of agreement obtained after removing the proportion of agreement that could be expected to occur by chance. Kappa is always less than or equal to 1. A value of 1 implies perfect agreement and values of less than 1 imply less than perfect agreement. Kappa coefficients ranged from 0.70 to 0.74, indicating good agreement [32]. Table 3 presents the results of the test – retest reliability of the Greek version of the Royal Free Interview for Spiritual and Religious Beliefs, while in table 4 the mean test-retest score, intra-class correlation coefficient (ICC), test-retest correlation (rho) and p value are presented. As shown in table 4, the Pearson correlation coefficient for the total test-retest score of the spiritual scale was 0.754 (p < 0.001).

**Table 3:** Test retest reliability of spiritual scale

**Questions with categorical responses**	**Kappa**	
	**Present study**	**King et al. 2001**

Q1. Belief system	0.73	0.79
Q12. Do you communicate with this power?	0.74	0.76
Q13. Do you think we exist in some form after death?	0.70	0.79
Q14. Have you ever had an intense experience?	0.72	0.78

**Table 4:** The mean test-retest score, intraclass correlation coefficient (ICC), test retest correlation (rho) and p value

**Scale item**	ICC*	**Mean (sd) score^+^**			**Rho**	**p**
				
		**Test**	**Retest**	**Difference**		
3	0.73	7.20 (2.07)	7.08 (2.24)	0.12 (0.17)	0.581	<.001
7	0.80	5.76 (2.87)	5.76 (2.77)	0 (0.10)	0.670	<.001
8	0.80	6.37 (2.66)	6.34 (2.61)	0.03 (0.05)	0.675	<.001
9	0.81	6.38 (2.61)	6.24 (2.58)	0.13 (0.03)	0.681	<.001
10	0.87	4.47 (2.92)	4.53 (2.96)	0.06 (0.04)	0.770	<.001
11	0.86	5.37 (3.10)	5.33 (3.09)	0.04 (0.01)	0.755	<.001
Total Score	0.86	35.55 (11.71)	35.28 (11.04)	0.27 (0.67)	0.754	<.001

*p < 0.001 for all ICCs, ^+^*t*-test for paired comparisons not significant.

**Figure 1:**
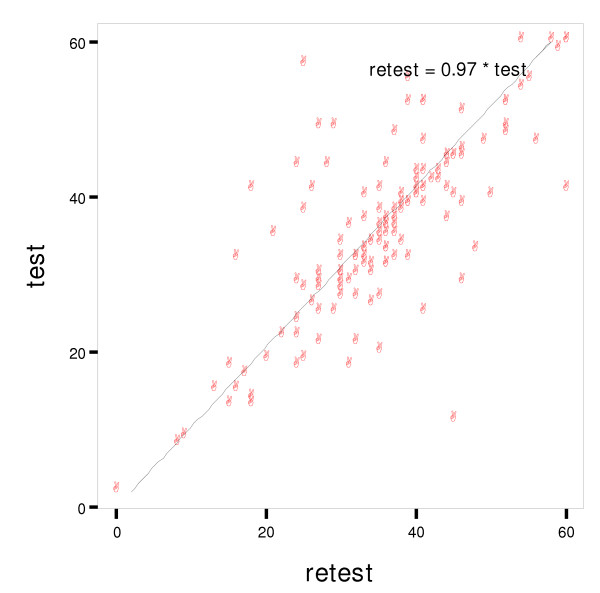
Bivariate scatterplot between the test and retest total scores of the spiritual scale

The bivariate scatterplot between the total test and retest scores of the spiritual scale are shown in figure 1. It gives a good visual picture of the relationship between the two variables and facilitates the interpretation of the regression model. As we see in figure 1, the points of test-retest plot are very close to or follow the regression line. This finding reasserts the test-retest reliability of the spiritual scale.

## Discussion

Judging from the results obtained the Greek version of the Royal Free Interview for Religious and Spiritual Beliefs proved to have satisfactory psychometric properties for a Greek population. The spiritual scale displayed good reliability, with sound internal consistency as assessed by coefficient α, and a degree of test-retest reliability similar to that reported by King et al. [24]. The excellent Pearson correlation coefficient for the test-retest of spiritual scale suggests that any repetition of the test would be likely to render the same results. The tool therefore proved to be reliable.

The intra-class correlation coefficient for continuous variables ranged from 0.73 to 0.86, and for total spiritual scale score it was 0.86. Coefficient kappa ranged from 0.70 to 0.74. In King et al., intra-class research correlation ranged from 0.72 to 0.89 and 0.94 for the total spiritual scale score. Coefficient kappa ranged from 0.76 to 0.79. These findings are evidence of cross-cultural test-retest agreement of scale items.

The majority of respondents replied that they believe in God and go to Church. Furthermore, the vast majority of participants (58.9%) reported both a religious and a spiritual belief. This is a constant finding of other research studies in the U.S.A. [37-39], with a percentage varying from 59% to 74%. It would seem that Greek respondents dissociate themselves from both spirituality and religion, and that they have a more traditional attitude toward religion. A similar attitude is mentioned by Streib in a research study on spirituality and religious orientation in adolescents in Germany [40]. However, we might interpret this evidence in the context of Christianity and with a view to the age structure of the respondents, who were very young. The low mean age of the sample may explain the distinction between spirituality and religiousness, as young people perceive the spiritual meaning of religiousness and distinguish it from church-related spirituality. In our sample, women were significantly more likely to hold a religious and/or spiritual view of life [24] and expressed a greater spirituality than men did.

The term "spirituality" is multidimensional, allowing for various interpretations with its many connotations and vague structure. Parker Palmer mentions that spiritual questions always revolve around angels or ethers or include the word God: *Spiritual questions are the kind that we and our patients ask every day of our lives, as we yearn to connect with the largeness of life: "Does my life have a meaning and a purpose?" "How do I deal with suffering?" "What is the real meaning of my life?" *[41] The last question accounts for the core meaning of the term spirituality for Greeks, as it includes all features that give meaning and purpose to life. All these approaches challenge the perceptual and conceptual framework of any instrument assessing spirituality and religiousness. The Greek version of the Royal Free Interview for Religious and Spiritual Beliefs captures all dimensions of this construct. Further evaluation of the scale might include a wider population, encompassing ethnic minorities and persons representing a greater variety of religions.

There are limits to the study to be discussed here. During its initial stage, we did not select a random sample. However, we decided to proceed with a convenient sample because it is very difficult to locate and approach certain groups of persons holding strong religious beliefs. Even if one succeeds in approaching them, it is difficult to persuade them to participate in such a study, since they tend to feel embarrassed and suspicious. Nevertheless, some attempts were made to approach persons belonging to Christian Orthodox groups, as it is generally believed that they have a stronger spiritual belief and commitment to Orthodox doctrine. Despite lengthy efforts, we did not achieve our objective. As a result, we approached not only Orthodox Christians – the vast majority of the Greek population – but also Roman Catholics and Protestants. A further concern was that due to language barriers, our sample consisted chiefly of persons fluent in spoken and written Greek. Despite these restrictions, we decided to proceed with checking the psychometric properties of our instrument, as we believed that this study could serve as a precursor for future research by contributing important insights to the psychometric properties of this instrument, i.e. pertaining to populations from minority groups and persons with a wider range of spiritual beliefs.

## Conclusion

In summary, the self-report version of the Royal Free Interview for Religious and Spiritual Beliefs appears to be a valid and a reliable test-retest measure of spirituality in a general Greek population. Future validation studies with multiple populations and a longitudinal design will be required in order to refine the instrument as an additional scale of spirituality.
